# *In Vitro* Antiplasmodial, Heme Polymerization, and Cytotoxicity of Hydroxyxanthone Derivatives

**DOI:** 10.1155/2021/8866681

**Published:** 2021-03-31

**Authors:** Mistika Zakiah, Rul Afiyah Syarif, Mustofa Mustofa, Jumina Jumina, Nela Fatmasari, Eti Nurwening Sholikhah

**Affiliations:** ^1^Department of Pharmacology and Therapy, Faculty of Medicine, Public Health and Nursing, Universitas Gadjah Mada, Yogyakarta 55281, Sekip Utara, Indonesia; ^2^Faculty of Medicine, Universitas Tanjungpura, Pontianak 78115, Indonesia; ^3^Department of Chemistry, Faculty of Mathematics and Natural Sciences, Universitas Gadjah Mada, Yogyakarta 55281, Sekip Utara, Indonesia

## Abstract

The previous study showed that xanthone had antiplasmodial activity. Xanthone, with additional hydroxyl groups, was synthesized to increase its antiplasmodial activity. One of the strategies to evaluate a compound that can be developed into an antimalarial drug is by testing its mechanism in inhibiting heme polymerization. In acidic condition, hematin can be polymerized to *β*-hematin *in vitro*, which is analog with hemozoin in *Plasmodium*. This study was conducted to evaluate the antiplasmodial activity of hydroxyxanthone derivative compounds on two strains of *Plasmodium falciparum* 3D-7 and FCR-3, to assess inhibition of heme polymerization activity and determine the selectivity of hydroxyxanthone derivative compounds. The antiplasmodial activity of each compound was tested on *Plasmodium falciparum* 3D-7 and FCR-3 with 72 hours incubation period, triplicated in three replications with the microscopic method. The compound that showed the best antiplasmodial activity underwent flow cytometry assay. Heme polymerization inhibition test was performed using the in vitro heme polymerization inhibition activity (HPIA) assay. The antiplasmodial activity and heme polymerization inhibition activity were expressed as the 50% inhibitory concentration (IC_50_). In vitro cytotoxicity was tested using the MTT assay method on Vero cell lines to determine its selectivity index. The results showed that among 5-hydroxyxanthone derivative compounds, the 1,6,8-trihydroxyxanthone had the best *in vitro* antiplasmodial activity on both 3D-7 and FCR-3 *Plasmodium falciparum* strains with IC_50_ values of 6.10 ± 2.01 and 6.76 ± 2.38 *μ*M, respectively. The 1,6,8-trihydroxyxanthone showed inhibition activity of heme polymerization with IC_50_ value of 2.854 mM and showed the high selectivity with selectivity index of 502.2–556.54. In conclusion, among 5-hydroxyxanthone derivatives tested, the 1,6,8-trihydroxyxantone showed the best antiplasmodial activity and has heme polymerization inhibition activity and high selectivity.

## 1. Introduction

Malaria is an infectious disease caused by a *Plasmodium* parasite transmitted through female *Anopheles* mosquito bites [1]. There are five types of *Plasmodium* species that cause malaria in humans: *Plasmodium falciparum, Plasmodium vivax, Plasmodium ovale, Plasmodium malariae,* and *Plasmodium knowlesi. Plasmodium falciparum* (*P. falciparum*) is a cause of malaria with severe symptoms which can lead to death [2].

One of the mechanisms of antimalarials is inhibiting the polymerization of heme. The polymerization of the heme is the process of changing free heme to hemozoin. One of the drugs that have a mechanism of action inhibiting the polymerization of heme is chloroquine [3, 4]. The formation of chloroquine and heme complexes can inhibit hemozoin formation [5]. The chloroquine target is to bind the heme. Free heme in *Plasmodium's* digestive vacuoles is toxic to cell membranes and *Plasmodium* proteolytic enzymes. The free heme is then polymerized by the *Plasmodium* into nontoxic hemozoin to protect the *Plasmodium* life. Hemozoin formation occurs only in the *Plasmodium* infection of the erythrocytic cycle. Hemozoin has a similar structure to *β*-hematin, while heme is similar to hematin. Therefore, by assessing this process, a compound can be developed into an antimalarial drug using the mechanism of action of heme polymerization. *In vitro* hematin can be polymerized into *β*-hematin in the acidic conditions, which has the same properties as existing hemozoin in the *Plasmodium* [6].

*Plasmodium falciparum* resistance to antimalarials is one of the factors of malaria treatment failure [7–9]. More advanced research is required to find new antimalarials. One of the strategies is to synthesize new compounds from the guiding compounds that have been known to have antiplasmodial activity. The determination of a compound to be used as a guide compound can be based on the resemblance of its chemical structure with other compounds that have been known to have high antiplasmodial activity [10].

One of the compounds that are promising to be developed as an antiplasmodial alternative is the xanthone derivative compound. Xanthone is a natural phenolic compound known to have antiplasmodial activity. Various studies have shown that the xanthone compounds of natural materials are proven to have the inhibition of *Plasmodium* growth [11, 12]. While encouraging as candidates, these potential antiplasmodials, derived from natural compounds, have a limited amount, so that their availability cannot be guaranteed. Therefore, to ensure the availability and sustainability of its production, it is necessary to develop a synthetic compound that can be remanufactured in large quantities. One study conducted by Amanatie et al. reported that the xanthone derived 2-hydroxyxanthone compound had an antiplasmodial activity with inhibitory concentration of 50% (IC_50_) of 4.385 *μ*g/mL. The study stated that the hydroxyl group influenced the high antiplasmodial activity of hydroxyxanthone in the xanthone framework. The addition of a hydroxyl group to the xanthone derivatives causes the antiplasmodial activity of the compound to be higher than the xanthone compound before it is tied to the hydroxyl group [13].

Some hydroxyxanthone derivative compounds have been synthesized by Fatmasari [14], however, their antiplasmodial activity is not known. Previous studies showed that an interaction occurs between the phenol compounds with the hematin electronic system. The phenol compound with a hydroxyl group can bind the heme iron [6]. The hydroxyl clusters that bind to hematin can form complexes that it will inhibit the formation of *β*-hematin *in vitro*. The inhibitory test of the polymerization of heme can be used to identify the mechanisms of action of antiplasmodials. Testing cytotoxicity on Vero cells can be conducted to evaluate the safety of hydroxyxanthone derivative compounds. To develop the hydroxyxanthone derivative compounds as malaria treatment, it is necessary to test their antiplasmodial activity, heme polymerization inhibitory activity, and cytotoxic effect on the Vero cells.

## 2. Materials and Methods

### 2.1. Testing Compounds and Plasmodium

Five hydroxyxanthone derivatives have been synthesized by Fatmasari [14], i.e., 1,6,8-trihydroxyxanthone (HX1); 1,6-dihydroxyxanthone (HX2); 1,5,6-trihydroxyxanthone (HX3); 1-hydroxy-5-chloroxanthone (HX4); and 1,6-dihydroxy-5-methylxanthone (HX5). The *Plasmodium falciparum* 3D-7 (chloroquine-sensitive strain) and FCR-3 (chloroquine-resistant strain) were obtained from laboratory collection of Department of Pharmacology and Therapy, Faculty of Medicine Public Health and Nursing, Universitas Gadjah Mada, Yogyakarta, Indonesia.

### 2.2. Plasmodium Culture

The test of antiplasmodial activity began with the culture of *P. falciparum* 3D-7 and FCR-3 using the modified Trager and Jensen method [15]. The plasmodium was cultured in human O red blood cells diluted to 3% haematocrit in RPMI 1640 medium complemented with 10% human O serum. The medium was made by adding 10.43 g of powder RPMI 1640, 6 g HEPES, 2 g NaHCO_3_, 25 mg gentamycin, and sterile distilled water up to 1 L. The medium pH was adjusted so that it reached ±7.2. It was sterilized using a 0.22 *μ*m filter and stored at 4°C. The complete plasmodium culture medium was made by adding human serum with a concentration of 10% in the medium. The plasmodium culture was incubated in candle jar in an incubator with a temperature of 37°C and was observed every 24 hours.

### 2.3. *In Vitro* Antiplasmodial Activity Assay

The plasmodium was synchronized to obtain the ring stage by adding 5% of D-sorbitol. The plasmodium was transferred from culture flask to a conical tube, and then it was centrifuged with a speed of 1000 rpm for 10 minutes. After the supernatant was disposed, the sterile 5% sorbitol was added and incubated for 10 minutes at a temperature of 37°C. The plasmodium suspension was centrifuged again; the supernatant was disposed and the plasmodium was washed by adding culture medium. Then, the plasmodium suspension was centrifuged again, and the supernatant was discarded, resulting in a plasmodium in ring stage only. The parasitemia was calculated from a thin blood preparation. The test used 1% parasitemia at 2% haematocrit in RPMI medium which complemented with 10% human O serum.

Each testing compound was dissolved in RPMI medium. The testing compound in various concentrations with volume of 100 *μ*L was incorporated into the 96-well microplate, and then 100 *μ*L of *Plasmodium* suspension was added. Each series of concentrations was replicated three times. The microplate was incubated at a temperature of 37°C for 72 hours. At the end of the incubation, a thin blood smear was made using 10% Giemsa stain and observed under a light microscope at 1000x magnification. The percentage of parasitemia (counting a minimum of 1,000 erythrocytes) was calculated from the preparation of thin blood and then used to calculate the percentage of plasmodium growth inhibition. As a control, plasmodium culture without any testing compounds was considered to have a growth of 100%. Antiplasmodial activity is expressed as IC_50_, which is the concentration of a compound that is required for 50% inhibition of plasmodium growth. The IC_50_ value was calculated by probit analysis using SPSS software (IBM Corp., Chicago). The lower the IC_50_ value obtained, the greater the antiplasmodial activity. The antiplasmodial activity was classified into 5 categories, i.e., excellent (IC50 < 1 *μ*M), good (IC50 1–20 *μ*M), moderate (IC50 20–100 *μ*M), low (IC50 100–200 *μ*M), and inactive (IC50 > 200 *μ*M) [16, 17].

### 2.4. Flow Cytometry Method

The *Plasmodium falciparum* strains of 3D-7 were used in this method. The plasmodium synchronized to obtain the ring stage by adding 5% of D-sorbitol. Plasmodium with 1% parasitemia was cultured in a 96-well microplate. The testing compounds were added in duplicate and incubated at 37°C for 72 hours. The samples were centrifuged with a speed of 1000 rpm for 10 minutes and washed twice in 100 *μ*L of phosphate-buffered saline (PBS). Samples were incubated with 50 *μ*L of 1 : 1000 SYBR green I and 20*μ*L of CD235A-PE for 15 minutes at room temperature. Cells were washed and resuspended in PBS. Data were obtained using a FACSCalibur with the acquisition of 1,00,000 events per sample. Initial gating was done with uninfected and unstained erythrocytes to account for erythrocyte autofluorescence. The control plasmodium-infected erythrocyte was referred as 100% growth to calculate the percentage of growth inhibition after treated with testing compound.

### 2.5. *In Vitro* Heme Polymerization Inhibitory Activity Assay

The heme polymerization inhibitory activity (HPIA) was conducted according to Basilico et al.'s modified method [6]. The 100 *μ*L of 1 mM hematin in 0.2 M NaOH was added into a microtube. Then, 50 *μ*L of testing compound at various concentrations (20.475; 10.238; 5.119; 2.580; 1.269 mM) was added in triplicates. Distilled water was used as a negative control. The 50 *μ*L solution of glacial acetic acid (pH 2.6) was added into the microtube to initiate the polymerization reaction and incubated at a temperature of 37°C for 24 hours. Microtubes were centrifuged at 8000 rpm for 10 minutes and the supernatant was discarded and then washed three times using the 200 *μ*L dimethyl sulfoxide (DMSO).Then, the deposition of the hematin crystal was dissolved with 200 *μ*L of 0.1 M NaOH and 100 *μ*L of solution was added into the 96-well microplate.

Absorbance was read using ELISA reader at *λ* 405 nm. A standard curve was made to illustrate the relationship between the concentration of hematin and its absorbance. The various concentrations of hematin (250; 125; 62.5; 31.25; 15.6; 7.8; and 3.9 mM) were used to make the standard curve. The heme polymerization inhibition was expressed as IC_50,_ which is the concentration of testing compounds that can inhibit 50% of the formation of *β*-hematin. The IC_50_ value was calculated by probit analysis using SPSS software (IBM Corp., Chicago). A compound shows to have heme polymerization inhibition activity in if it has an IC_50_ value lower than the IC_50_ value of chloroquine as in reference (37.5 mM) [17].

### 2.6. *In vitro* Cytotoxicity Test on Vero Cell

The *in vitro* cytotoxicity test was conducted using the 3-(4,5-dimethylthiazol-2-yl)2,5-diphenyltetrazolium bromide (MTT) assay method on Vero cell line culture using the Anderson et al.'s modified method [18].

### 2.7. Vero Cell Line Culture

The cell was removed from the cryo medium. Then, the cell was thawed at 37°C. The liquid cell was subsequently transferred to the tube and a culture medium was added to a volume of 10 mL. Then, the suspension was centrifuged for 15 minutes so that it would form cell pellets. The supernatant was removed and 1 mL of culture medium was added to the cell pellets. Finally, the suspension was transferred to the Petri dish and incubated in the incubator with 5% of CO_2_ at 37°C.

### 2.8. Cell Harvesting

The medium of Vero cell culture was removed, and then the cell was washed using a PBS solution with a volume of PBS ± 1/2 of the initial medium volume. This step was done twice and 1 mL of trypsin 0.25% was added. Subsequently, the cell was incubated in the CO_2_ incubator at 37°C for 3 minutes. Then, the cell was resuspended using 2 mL of culture medium and PBS solution was added up to 10 mL. The suspension was centrifuged for 15 minutes so that it formed pellets. Pellets were added with 1 mL of culture medium and resuspended. Take 10 *μ*L of the cell suspension and density of cells was calculated.

Then, the cell was added with culture medium to 10 mL. A total of 100 *μ*L of such suspensions was transferred into each well of the 96-well microplate, but three of the wells were emptied as medium controls. Subsequently, the microplate was incubated in a 5% CO_2_ incubator at 37°C.

### 2.9. Preparation of Testing Compounds

Each hydroxyxanthone derivative compound was dissolved in DMSO, and a variety of concentrations were prepared in the medium. Each concentration of the testing compound was added into the 96-well microplate triplicately. Cell control and medium control were not given a testing compound solution. The medium control remained blank, while the cell control contained culture medium only. Chloroquine was used as a positive control. Then, the cell was incubated in a 5% CO_2_ incubator at 37°C for 24 hours.

### 2.10. MTT and SDS Addition

After the cell was incubated for 24 hours, the medium was discarded and washed with PBS. Then, each well was added with 100 *μ*L of 5 mg/mL of MTT solution. The 96-well microplate was incubated again in the incubator of CO_2_ at 37°C for 4 hours. Then, 100 *μ*L of 10% of SDS in 0.01 MHCl was added. The cells were placed in a dark room at room temperature for 24 hours.

### 2.11. Measurement of the Absorbance and IC_50_ Value

The absorbance was measured using the enzyme-linked immunosorbent assay (ELISA) reader at 595 nm wavelength. The cytotoxicity test on the Vero cells was done by calculating the percentage of Vero cell inhibition of the absorption value of the test compound, cell control, and medium control. The cytotoxic effect was expressed as IC_50_. The IC_50_ value was obtained from the probit analysis by using SPSS software (IBM Corp., Chicago). The degree of selectivity was expressed as a selectivity index. The selectivity index was obtained from the ratio between the IC_50_ cytotoxic effect on Vero cells and IC_50_ antiplasmodial activity on *Plasmodium* [19]. A compound is said to be safe if the value of the selectivity index is >10 [20].

## 3. Results

### 3.1. *In Vitro* Antiplasmodial Activity Assay

The increasing concentrations of testing compounds showed increasing the percentage of *Plasmodium* growth inhibition. The percentages of *Plasmodium* growth inhibition of hydroxyxanthone derivatives and chloroquine on *P. falciparum* 3D-7 and FCR-3 are presented in Table 1. The IC_50_ values of hydroxyxanthone derivatives on *P. falciparum* 3D-7 and FCR-3 are presented in Table 2. The lowest IC_50_ value of the hydroxyxanthone derivative compound on *P. falciparum* 3D-7 6.10 ± 2.01 *μ*M was found in HX1. In contrast, the highest IC_50_ value of hydroxyxanthone derivative compounds on *P. falciparum* strain 3D7 85.30 ± 4.87 *μ*M was found in HX4. The lowest IC_50_ value of the hydroxyxanthone derivative compound with the microscopic method on *P. falciparum* FCR-3 was also found in HX1 (6.76 ± 2.38 *μ*M) while the highest IC_50_ value of hydroxyxanthone derivatives on *P. falciparum* FCR-3 was 89.85 ± 17.69 *μ*M found in HX4. These results showed that the HX1 showed good antiplasmodial activity, whereas HX2, HX3, HX4, and HX5 had moderate antiplasmodial activity on *P. falciparum* 3D-7 and FCR-3. Chloroquine as positive control had IC_50_ value 0.01 ± 0.001 on *P. falciparum* strain 3D-7 and 0.11 ± 0.052 on *P. falciparum* strain FCR-3.

### 3.2. Flow Cytometry Method

**Figure 1:**
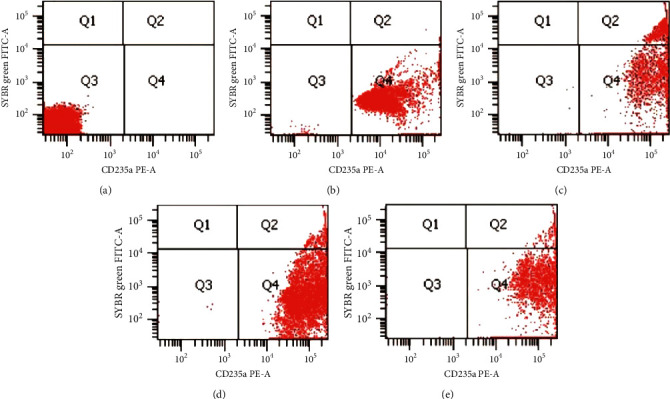
The flow cytometric analysis diagram of compound HX1 on *Plasmodium falciparum* 3D-7 with 72 hours incubation. (a) Normal unstained erythrocytes. (b) Normal stained red erythrocytes (uninfected). (c) *P. falciparum*-infected erythrocytes control. (d) *P. falciparum*-infected erythrocytes with HX1 (concentration of 102.38 *μ*M). (e) *P. falciparum*-infected erythrocytes with HX1 (concentration of 204.75 *μ*M). The diagram was divided into four regions: Q1 (FITC/PE. ±); Q2 (FITC/PE. +/+); Q3 (FITC/PE. -/-); Q4 (FITC/PE. ±).

In flow cytometry assay, the compound HX1 with an optimal concentration of 102.38 *μ*M showed inhibition activity of *P. falciparum* 3D-7 growth. The higher concentration of compound HX1 with a concentration of 204.75 *μ*M showed the higher inhibition activity by more than 90%. The flow cytometric analysis diagram of compound HX1 on *P. falciparum* 3D-7 is presented in Figure 1.

### 3.3. *In Vitro* Heme Polymerization Inhibitory Activity Assay

The results showed that the higher the concentration of the HX1, the lower the amount of *β*-hematin formed, and the higher the inhibitory percentage of *β*-hematin. The HX1 at concentration of 20.475 mM were able to inhibit the polymerization of heme with IC_50_ value which is 2.854 mM. In comparison, the IC_50_ value of chloroquine as the positive control is 1.478 mM. The IC_50_ values of 1,6,8-trihydroxyxanthone (HX1) compound and chloroquine in inhibiting the formation of the *β*-hematin are presented in Table 3.

### 3.4. *In Vitro* Cytotoxicity Test on Vero Cell

Among 5-hydroxyxanthone compounds tested, the compound HX1 showed the highest IC_50_ value in *in vitro* cytotoxicity test on Vero cells (3394.90 ± 435.44 *μ*M). Compound HX1 showed the highest value of the selectivity index (cytotoxicity/antiplasmodium ratio on *P. falciparum* strain 3D-7 and FCR-3) with range of 502.20–556.54. The IC_50_ values of hydroxyxanthone compounds on Vero cells are presented in Table 4. The selectivity index of hydroxyxanthone compounds and chloroquine is presented in Table 5.

## 4. Discussion

### 4.1. *In Vitro* Antiplasmodial Activity of Hydroxyxanthone Derivative Compounds

The study showed that the hydroxyxanthone derivative compounds tested showed different levels of antiplasmodial activity. Compound 1,6,8-trihydroxyxanthone (HX1) showed the best antiplasmodial activity among five hydroxyxanthone compounds tested using microscopic method. The category of antiplasmodial activity of the hydroxyxanthone compound was determined according to Batista et al.'s criteria [17] that divide a compound into five categories, i.e., very good(IC_50_ < 1 *μ*M); good (IC_50_ 1–20 *μ*M); moderate (IC_50_ 20–100 *μ*M); low (IC_50_ 100–200 *μ*M); and inactive (IC_50_ > 200 *μ*M). *In vitro* antiplasmodial activity assay conducted on *Plasmodium* strains of 3D-7 and FCR-3 showed an IC_50_ value of 6.10 ± 2.01 *μ*M and 6.76 ± 2.38 *μ*M for the 1,6,8 -trihydroxyxanthone(HX1), which showed that this compound has a good antiplasmodial activity. The flow cytometry analysis with SYBR green I and CD235A demonstrated a clear separation of infected erythrocytes with *P. falciparum* and uninfected erythrocytes (Figure 1). This method identifies *P. falciparum* based on nucleic acid contents [21].

Based on its structure, HX1 consists of a hydroxyl group, C-H, aromatic (C=C), cluster that characterizes the xanthone compound, which is the top of the carbonyl group (C=O) (carbonyl number 9) and the ether group (C-O-C) (Carbon numbers 4a and 4b) [14]. The hydroxyl of the xanthone compound can bind to the carboxyl heme group, an aromatic role in the stability of the hydroxyxanthone-heme complex, and the carbonyl group of the xanthone compound can bind with the heme iron so that the hydroxyxanthone-heme complex is formed [22]. The interaction with the free heme is most likely to contribute to the mechanism of detoxification of the heme. It is one of the known mechanisms of antiplasmodial action.

Amanatie reported the activity of the hydroxyxanthone compound, which had a distinct number of hydroxyl clusters in the *P. falciparum,* and identified that the 2-hydroxyxanthone compound had an antiplasmodial activity on *P. falciparum* strain 3D-7 with IC_50_ 20.69 *μ*M, whereas IC_50_ value of 2,7-dihydroxyxanthone was 1.342 *μ*M [13]. The more the hydroxyl clusters on the xanthone framework, the better the antiplasmodial activity [23]. These results are in line with the results in this study that found HX1 and HX3 have lower IC_50_ values which showed that these compounds are more active than HX2, HX5, and HX4 which have fewer number of hydroxyl clusters. In addition to the number of hydroxyl groups, differences in antiplasmodial activity can be due to differences in substituent and position of the hydroxyl group in the hydroxyxanthone derivatives. The HX5 compound has a methyl group, which is an electron donor. Still, the cluster's electron donor strength was weaker than the hydroxyl group, and the HX4 compound was an electron puller, chlorosubstituent [14]. The study showed that the addition of methyl and chlorogroups to the carbon position of 5-hydroxyxanthone compounds had not been able to provide better activity shown in HX5 and HX4 compounds, which had a higher IC_50_ value than HX2.

Previous study on the hydroxyxanthone compounds reported that xanthone compounds substituted hydroxyl clusters at carbon positions 2, 3, 4, 5, and 6 had *in vitro* potential of antiplasmodial [13]. The same study was reported by Ignatuschenko et al. [11], which found xanthone, with the addition of 1-hydroxyl group at position 4 or 5, and had moderate antiplasmodial activity. In contrast, xanthone with the addition of hydroxyl groups in both locations 4 and 5 have more active antiplasmodial activity. The data were in line with this study, i.e., the addition of a hydroxyl group in carbon position 6 in compounds HX1, HX3, HX2, and HX5 which had lower IC_50_ value than the HX4 that has a hydroxyl group in the position of carbon 6.

### 4.2. Polymerization Inhibitory Activity of Hydroxyxanthone Derivative Compounds

When *Plasmodium* infects human erythrocytes, it brings hemoglobin from erythrocytes to the *Plasmodium*'s digestive vacuoles. *Plasmodium* has acid pH through the process of pinocytosis [24]. The hemoglobin is oxidized to methemoglobin, which is hydrolyzed into free heme and globin by the aspartic proteases (plasmepsin I, II, IV and histo-aspartic protease). Globin is hydrolyzed to peptides by cysteine proteases (falcipain) and zinc-containing metallopeptidase. Then, peptides are hydrolyzed to amino acids by cytosol exopeptidase, which will be used by *Plasmodium* for protein synthesis from the vacuole membranes to the *Plasmodium* cytoplasm [25, 26].

The result of the degradation of hemoglobin is that the free heme is toxic to the *Plasmodium* because it produces reactive oxygen species (ROS) and induces oxidative stress, which leads to cell lysis and the parasite's death [27]. Therefore, *Plasmodium* resolves the toxicity of free heme with the heme detoxification system [28]. The primary heme detoxification system is the formation of hemozoin, which occurs in the *Plasmodium*'s digestive vacuole. In contrast, the secondary heme detoxification system, via H_2_O_2_ and glutathione (GSH) and with a protein-heme binding, occurs in the cytosol. In the *Plasmodium*'s digestive vacuole, 30–50% of the free heme will be converted to nontoxic hemozoin while the other free heme passes through the *Plasmodium*'s digestive vacuole to the *Plasmodium* cytosol then neutralized by GSH, H_2_O_2_, and heme-protein bond [29].

The free heme that is the heme monomer will be converted into the form of a polymer heme (hemozoin) by the enzyme of heme polymerase. Hemozoin (malaria pigment) is stable and nontoxic. The heme polymer is interconnected with the iron-carboxylase bond that connects the Ferri center of one heme with the side chain propionate of the other heme. Hemozoin formation is antimalarials' target, such as chloroquine. The target activity is to inhibit the polymerization of heme [10, 30, 31].

The HX1 compound, which had the best antiplasmodial activity in this research, was tested *in vitro* for its heme polymerization inhibitory activity. The test involves a spontaneous hemozoin formation mechanism (*β*-hematin). Hematin was used in this assay because it has a similar structure to the free heme, whereas to mimic the model of the *Plasmodium*'s digestive vacuoles, which has acidic pH and initiates the reaction of the formation of *β*-hematin; glacial acetic acid was administered. In acidic environments, the hematin will polymerize into the crystal *β*-hematin [24].

The lesser the amount of *β*-hematin formed, the more inhibition of the polymerization of heme. This test suggested that the administration of HX1 can inhibit the formation of *β*-hematin. The higher the concentration of compounds, the greater the inhibitory percentage of *β*-hematin will be. The HX1 compound can inhibit the formation of *β*-hematin with an IC_50_ value of 2.854 mM. Xanthone's molecular characteristics make it insoluble in water, as well as having a high solubility to organic solvents. Nonpolluting characters cause most xanthone compounds to have some biological effects that can affect the membrane [32].

Chloroquine will diffuse the *Plasmodium*'s digestive vacuole membrane lipid bilayer. Inside the *Plasmodium* vacuole, chloroquine is protonated and cannot be diffused out so that chloroquine accumulates in the *Plasmodium*'s digestive vacuole. Chloroquine then binds to the heme forming a stable chloroquine-heme complex that will inhibit the polymerization of the heme resulting in disruption of the structure and damage of the digestive vacuole as well as the *Plasmodium* death. Consistent results are demonstrated from the activity structure profile of the 4,5-dihydroxyxanthone (45X2) compound that has *in vitro* antiplasmodial activity [11, 24].

Spectroscopic nuclear magnetic resonance (NMR) research reported that the structure of hydroxyxanthone (45X2)-heme complex was formed from an interaction between the iron heme and the carbonyl group in the xanthone compound. Two aromatic systems contribute to the stability of the heme-45X2 complex along with the hydrogen bond between the hydroxyl clusters on the xanthone compound with the carboxyl groups in the heme. The bond can depict a 45X2 model with *μ*-oxo dimer hematin. The study expressed a 45X2 bond affinity with heme almost identical to the heme-antimalaria complex, such as chloroquine and quinine. Interaction stoichiometry 45X2-heme showed two units of heme bonded with one 45X2 molecule [22]. The data gave an overview of how the hydroxyxanthone compound can bind to the heme.

Earlier research conducted with H-NMR spectroscopy also showed the interaction of chloroquine with hematin. The study reported the interaction of the chloroquine molecule that binds four hematin molecules. Chloroquine binds mainly with the form of *μ*-oxodimerhematin. The structure and stability of the *μ*-oxodimerhematin obstruct the formation of hemozoin. Its quinoline derivatives that bond to hematin can stabilize hematin in the form of the *μ*-oxo dimer, thereby reducing the amount of hematin monomer to be combined into hemozoin and inhibit the polymerization of hematin [33]. However, there are aminoquinoline derivatives that interact with hematin but do not impede hematin polymerization. Thus, binding of compounds with hematin does not necessarily cause inhibition of hematin polymerization. Inhibitory polymerization of heme is not only determined from the ability of the compound in binding heme but its expertise in inhibiting heme polymer bonding with other heme [31]. The displacement of hematin can be caused by bonding with unstable hematin, such as a particular cluster that is at position 7 of the quinoline derivatives ring which has a firm bond with hematin. Still, when the group is moved at location six on a quinoline derivatives ring, the bond with hematin becomes unstable and cannot inhibit the polymerization of hematin [24].

Heme polymerization inhibitory activity (HPIA) assay showed that HX1 had low IC_50_ (2.854 mM), as did chloroquine (1.478 mM). Chloroquine had a potential antiplasmodial activity with low IC_50_ value on *Plasmodium* 3D-7 and FCR-3 (0.001 and 0.11 *μ*M, respectively). The HX1 had less active antiplasmodial activity than chloroquine with IC_50_ value on *Plasmodium* 3D-7 and FCR-3 (6.10 and 6.76 *μ*M, respectively). The data indicated there was a correlation between inhibitory polymerization of heme with antiplasmodial activity. However, these results are not necessarily linear. Ignatuschenko et al. stated that there were differences in the antiplasmodial activity and heme polymerization inhibitory activity obtained from the derivatives of xanthone, namely, pentametoxyxanthone and pentaacetylxanthone. They were inactive in the heme polymerization inhibitory but had a potential or excellent antiplasmodial ability [11]. These data indicated that there was no direct correlation between heme polymerization inhibitory activity and antiplasmodial activity. Compounds that can inhibit the heme polymerization do not necessarily have good antiplasmodial activity. Compounds that have good antiplasmodial activity not necessarily inhibit the heme polymerization.

Some theories describe other mechanisms of action of antiplasmodial through the pathway of glycolysis. It inhibits the lactate dehydrogenase (LDH) enzyme and reduces adenosine triphosphate (ATP) production, which can a cause of *Plasmodium* death. Besides, the different mechanisms of action can be through DNA replication pathways by inhibiting the topoisomerase II enzyme, which will eventually lead to *Plasmodium* death [34, 35].

### 4.3. Cytotoxicity of Hydroxyxanthone Derivatives on Vero Cells

This cytotoxicity test was performed to determine the safety of hydroxyxanthone compounds on normal cells (Vero cells). The Vero cell culture which given the hydroxyxanthone compound indicates that the higher the concentration given, the higher the percentage of growth inhibition of Vero cells and becomes more toxic to the cells depending on the concentration.

Cytotoxicity of compounds HX1, HX2, HX3, HX4, and HX5 on the Vero cell was shown from the IC_50_ values (3283.72; 389.66; 230.28; 334.90; 458.94 *μ*M or 801.88; 88.92; 56.23; 82.60; 111.17 *μ*g/mL, respectively). The five hydroxyxanthone compounds have weak cytotoxic effects (IC50 > 30 *μ*g/mL) on the Vero cell [36]. The same results were presented in the research conducted by Fatmasari [14], showing weak cytotoxicity of compounds HX1, HX2, HX3, HX4, and HX5 on MCF-7, WIDR, and Hela cell lines.

*Insilico* interactions between the HX1 compound with the topoisomerase enzyme II have been conducted through the molecular docking process. The process of molecular docking involves the prediction of conformation and orientation of the ligands with binding sites (binding side) of the target protein. Compound or ligand serves as a complementary pair of proteins, while protein acts as a receptor. Mitoxantrone (MIX) is a ligand that has a basic frame work similar to xanthone found in the enzyme topoisomerase II. Also, both have a match of shape and volume on the active side then interaction ligand and protein occur. The lower the energy value, the more stable the ligand so that the interaction produced with protein is stronger. Thus, the presence of a variety of strong interactions can cause the performance of the topoisomerase II enzyme. The enzyme is found in viable cells and serves to improve the structure of damaged DNA. When the enzyme function is interrupted, the process of replication and transcription of DNA into RNA cannot take place so that the cell will experience apoptosis. The process is also one of the mechanisms of action from antiplasmodials, such as chloroquine.

The result of the molecular docking indicates the interaction of the DNA chains, namely, hydrogen interactions (DG C:13) and pi-pistacked (DA f:12 and DC c:8), with the binding affinity value −8.0 kcal/mol. However, the *in vitro* cytotoxicity test showing HX1 had a weak cytotoxic activity on MCF-7, WIDR, and Hela cell lines with IC_50_ 45; 61.9; 67.7 *μ*g/mL. These results demonstrated that the mechanism of action of the HX1 compound might also work in inhibition of the topoisomerase enzyme II. To determine the presence of the bond with DNA *in vitro* can be tested with DNA-methyl green assay with spectrophotometry [37].

The selectivity of a compound is crucial in the development of new drugs. The compound is said to be selective if the selectivity index is more than 10 and has a good antiplasmodial effect, but is not toxic to the host. The selectivity index was obtained from the ratio between (IC_50_ of cytotoxic effect on Vero cells) and (IC_50_ of antiplasmodial activity on *P. falciparum* 3D-7 and FCR-3). The HX1 has an index of selectivity >10, which means it is safe for the host so that it is potentially to be developed as an antiplasmodial.

## 5. Conclusions

Among 5-hydroxyxanthone derivatives tested, the 1,6,8-trihydroxyxantone showed the best antiplasmodial activity and has heme polymerization inhibition activity and high selectivity.

## Figures and Tables

**Table 1:** The percentage of Plasmodium growth inhibition of hydroxyxanthone derivatives and chloroquine on *P. falciparum* 3D-7 and FCR-3.

Compound	Concentration (*μ*M)	Growth inhibition of *P. falciparum* (%)	
		Strain 3D-7	Strain FCR-3
1,6,8*-*Trihydroxyxanthone (HX1)	102.38	75.864 ± 0.471	73.671 ± 4.277
	51.19	69.033 ± 0.888	64.446 ± 0.671
	25.59	64.726 ± 0.861	60.171 ± 2.739
	12.8	61.479 ± 0.710	55.160 ± 3.588
	6.39	47.405 ± 5.857	50.894 ± 2.308

1,6*-*Dihidroxyxanthone (HX2)	876.42	90.560 ± 0.665	87.623 ± 2.239
	438.21	73.618 ± 0.713	74.226 ± 1.318
	219.11	67.164 ± 0.601	67.144 ± 2.757
	109.55	54.805 ± 2.433	50.248 ± 2.629
	54.78	49.172 ± 1.606	47.192 ± 0.687

1,5,6*-*Trihydroxyxanthone (HX3)	409.5	87.438 ± 1.154	82.978 ± 1.396
	204.75	80.923 ± 0.679	71.858 ± 7.842
	102.38	68.886 ± 0.151	51.920 ± 0.466
	51.19	63.400 ± 0.421	43.133 ± 0.126
	25.59	47.295 ± 0.611	38.265 ± 1.186

1*-*Hydroxy-5-chloroxanthone (HX4)	810.86	74.002 ± 0.923	72.933 ± 1.345
	405.43	64.513 ± 0.418	61.202 ± 3.024
	202.71	57.136 ± 1.574	56.371 ± 3.792
	101.36	50.963 ± 0.361	49.754 ± 3.361
	50.68	46.473 ± 0.800	47.388 ± 0.736

1,6-Dihydroxy-5-methylxanthone (HX5)	330.25	93.288 ± 0.956	84.664 ± 0.902
	165.13	75.364 ± 1.287	73.462 ± 1.596
	82.56	54.180 ± 1.574	46.519 ± 0.816
	41.28	45.041 ± 0.563	41.140 ± 0.396
	20.64	37.886 ± 0.441	33.632 ± 0.327

Chloroquine	0.037	97.766 ± 0.690	84.189 ± 2.449
	0.029	97.490 ± 0.517	82.599 ± 2.945
	0.023	96.581 ± 1.330	79.923 ± 3.863
	0.016	96.354 ± 1.131	78.570 ± 4.507
	0.008	54.415 ± 12.42	18.748 ± 9.233

**Table 2:** The IC_50_ values of hydroxyxanthone derivatives on *Plasmodium falciparum* 3D-7 and FCR-3 after 72 hours incubation period using microscopic method.

Compound	Structure	IC_50_ (*μ*M) on *P. falciparum* 3D-7	IC_50_ (*μ*M) on *P. falciparum* FCR-3
1,6,8-Trihydroxyxanthone (HX1)	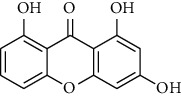	6.10 ± 2.01	6.76 ± 2.38
1,6-Dihydroxyxanthone (HX2)	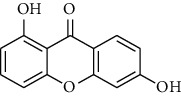	71.78 ± 0.31	81.77 ± 5.78
1,5,6-Trihydroxyxanthone (HX3)	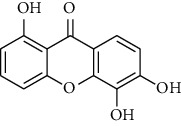	27.64 ± 0.19	64.09 ± 5.08
1-Hydroxy-5-chloroxanthone (HX4)	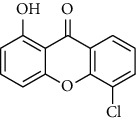	85,30 ± 0.87	89.85 ± 7.69
1,6-Dihydroxy-5-methylxanthone (HX5)	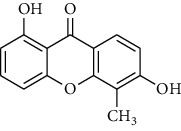	46.69 ± 0.29	59.73 ± 0.78
Chloroquine	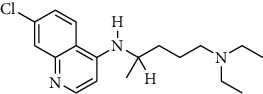	0.01 ± 0.001	0.11 ± 0.052

**Table 3:** The IC_50_ value of 1,6,8-trihydroxyxanthone (HX1) compound and chloroquine on the formation of the *β*-hematin using method of HPIA.

Compounds	Concentration (mM)	*β*-Hematin (mM)	Inhibition of *β*-hematin (%)	IC_50_ (mM)
HX1	20.475	9.684	91.171	2.854
	10.238	14.376	86.893	
	5.119	54.448	50.359	
	2.580	55.776	49.149	
	1.269	72.500	33.901	

*Chloroquine*	9.692	12.500	88.604	1.478
	4.846	22.040	79.906	
	2.423	47.845	56.379	
	1.221	57.787	47.315	
	0.601	77.787	29.080	

**Table 4:** The IC_50_ values of hydroxyxanthone compounds on Vero cell after 24 hours incubation using the MTT assay method.

Compounds	Concentration (*μ*M)	Vero cell growth inhibition (%) after 24 hours incubation	IC_50_ (*µ*M)

HX1	8190.01	89.43 ± 1.90	3394.90 ± 435.44
	4095.00	45.86 ± 5.51	
	2047.50	27.96 ± 5.69	
	1023.75	15.89 ± 8.24	
	511.88	−4.13 ± 8.21	
	255.94	−50.32 ± 17.18	

HX2	1734.91	86.09	308.32
	867.45	67.03	
	433.73	59.75	
	216.86	38.93	
	108.26	31.75	
	54.13	15.63	

HX3	2047.50	88.86	224.45
	1023.75	79.92	
	511.88	68.04	
	255.94	55.70	
	127.76	39.58	
	63.88	20.70	

HX4	2027.14	89.96	330.68
	1013.57	68.87	
	506.78	54.41	
	253.39	43.17	
	126.49	32.49	
	63.25	19.04	

HX5	2064.07	88.76	417.57
	1032.03	68.13	
	516.02	48.33	
	258.01	37.83	
	128.80	27.88	
	64.40	−5.19	

**Table 5:** The selectivity index (ratio IC_50_ on Vero cell/IC_50_ on plasmodium) of hydroxyxanthone compounds and chloroquine.

Compounds	IC_50_ on Vero cell (*μ*M)	Selectivity index	
		3D-7	FCR-3
HX1	3394.90	556.54	502.20
HX2	308.32	4.30	3.77
HX3	224.45	8.12	3.50
HX4	330.68	3.88	3.68
HX5	417.57	8.94	6.99
Chloroquine	2216.92	221692	20153.82

## Data Availability

The data used to support the findings of this study are available from the corresponding author upon request.
